# *MatchMaps*: non-isomorphous difference maps for X-ray crystallography

**DOI:** 10.1107/S1600576724003510

**Published:** 2024-05-17

**Authors:** Dennis E. Brookner, Doeke R. Hekstra

**Affiliations:** a Department of Molecular and Cellular Biology, Harvard University, Cambridge, Massachusetts, USA; b School of Engineering and Applied Sciences, Harvard University, Cambridge, Massachusetts, USA; Oak Ridge National Laboratory, USA; North Carolina State University, USA

**Keywords:** X-ray crystallography, open-source software, difference maps

## Abstract

Conformational change mediates the biological functions of macromolecules. Crystallographic measurements can map these changes with extraordinary sensitivity as a function of mutations, ligands and time. A popular method for detecting structural differences between crystallographic data sets is the isomorphous difference map. These maps combine the phases of a chosen reference state with the observed changes in structure factor amplitudes to yield a map of changes in electron density. Such maps are much more sensitive to conformational change than structure refinement is, and are unbiased in the sense that observed differences do not depend on refinement of the perturbed state. However, even modest changes in unit-cell properties can render isomorphous difference maps useless. This is unnecessary. Described here is a generalized procedure for calculating observed difference maps that retains the high sensitivity to conformational change and avoids structure refinement of the perturbed state. This procedure is implemented in an open-source Python package, *MatchMaps*, that can be run in any software environment supporting *PHENIX* [Liebschner *et al.* (2019). *Acta Cryst.* D**75**, 861–877] and *CCP4* [Agirre *et al.* (2023). *Acta Cryst.* D**79**, 449–461]. Worked examples show that *MatchMaps* ‘rescues’ observed difference electron-density maps for poorly isomorphous crystals, corrects artifacts in nominally isomorphous difference maps, and extends to detecting differences across copies within the asymmetric unit or across altogether different crystal forms.

## Abstract 2

*MatchMaps* is a generalization of the isomorphous difference map allowing for computation of difference maps between poorly isomorphous and non-isomorphous pairs of crystallographic data sets. *MatchMaps* is implemented as a simple-to-use Python-based command-line interface.

## 1. Introduction

X-ray crystallography provides a powerful method for characterizing the changes in protein structure caused by a perturbation (Hekstra *et al.*, 2016 ▸; Keedy *et al.*, 2018 ▸; Bhabha *et al.*, 2015 ▸; Brändén & Neutze, 2021 ▸). For significant structural changes, it is usually sufficient to refine separate structural models for each data set and draw comparisons between the refined structures. However, for many conformational changes, coordinate-based comparisons are inaccurate and insensitive.

In crystallography, electron density is not observed directly. Rather, one observes a diffraction pattern consisting of reflections with intensities proportional to the squared amplitudes of the structure factors – the Fourier components of the electron density. Unfortunately, the phases of these structure factors are not observable. These phases correspond in real space to shifts of the sinusoidal waves that add up to an electron-density pattern. Accordingly, phases are usually calculated from a refined model. Since phases have a strong effect on the map appearance (Read, 1986 ▸), naïve electron-density maps calculated using observed amplitudes and model-based phases will tend to resemble the model, a phenomenon known as model bias.

Conformational changes in crystallography, and especially in room-temperature or time-resolved crystallography, are often detected via an isomorphous difference map (Rould & Carter, 2003 ▸). Such a map is computed by combining differences in observed structure factor amplitudes with a single set of phases. The phases are usually derived from a model for one of the two states, chosen as a reference. Thus, the difference density Δρ(**x**) is approximated as 

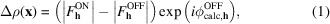

where 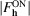 and 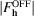 are sets of observed structure factor amplitudes from the ON (perturbed) and OFF (reference) data sets, respectively, 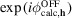 is a set of calculated structure factor phases derived from a structural model of the OFF data, **h** is shorthand for the triplet of Miller indices (*h*, *k*, *l*) and **x** is shorthand for the real-space fractional coordinates (*x*, *y*, *z*). Crucially, therefore, isomorphous difference maps do not include any information derived from modeling of the ON structure. Any difference electron density relating to the ON data relative to the OFF data (*e.g.* positive difference density for a bound ligand) is thus guaranteed not to be biased by previous modeling of the ON state. Unfortunately, interesting conformational changes often slightly alter the packing of molecules in the unit cell, which can manifest as changes in unit-cell dimensions. Unit-cell constants are also sensitive to temperature (Fraser *et al.*, 2011 ▸), radiation damage (Ravelli & McSweeney, 2000 ▸), pressure (Barstow *et al.*, 2008 ▸) and humidity (Farley *et al.*, 2014 ▸), meaning that even data collected on the same crystal may not be quite isomorphous.

In this contribution, we will illustrate the consequences of deviations from perfect isomorphism, introduce an approach to the calculation of difference maps without perfect iso­mor­phism, and describe examples of the application of the software implementing this approach (*MatchMaps*) to a number of typical use cases. We find the *MatchMaps* approach to be also applicable to molecules related by non-crystallographic symmetry and to molecules crystallized in altogether different crystal forms.

### 1.1. Implications of isomorphism

**Figure 1:**
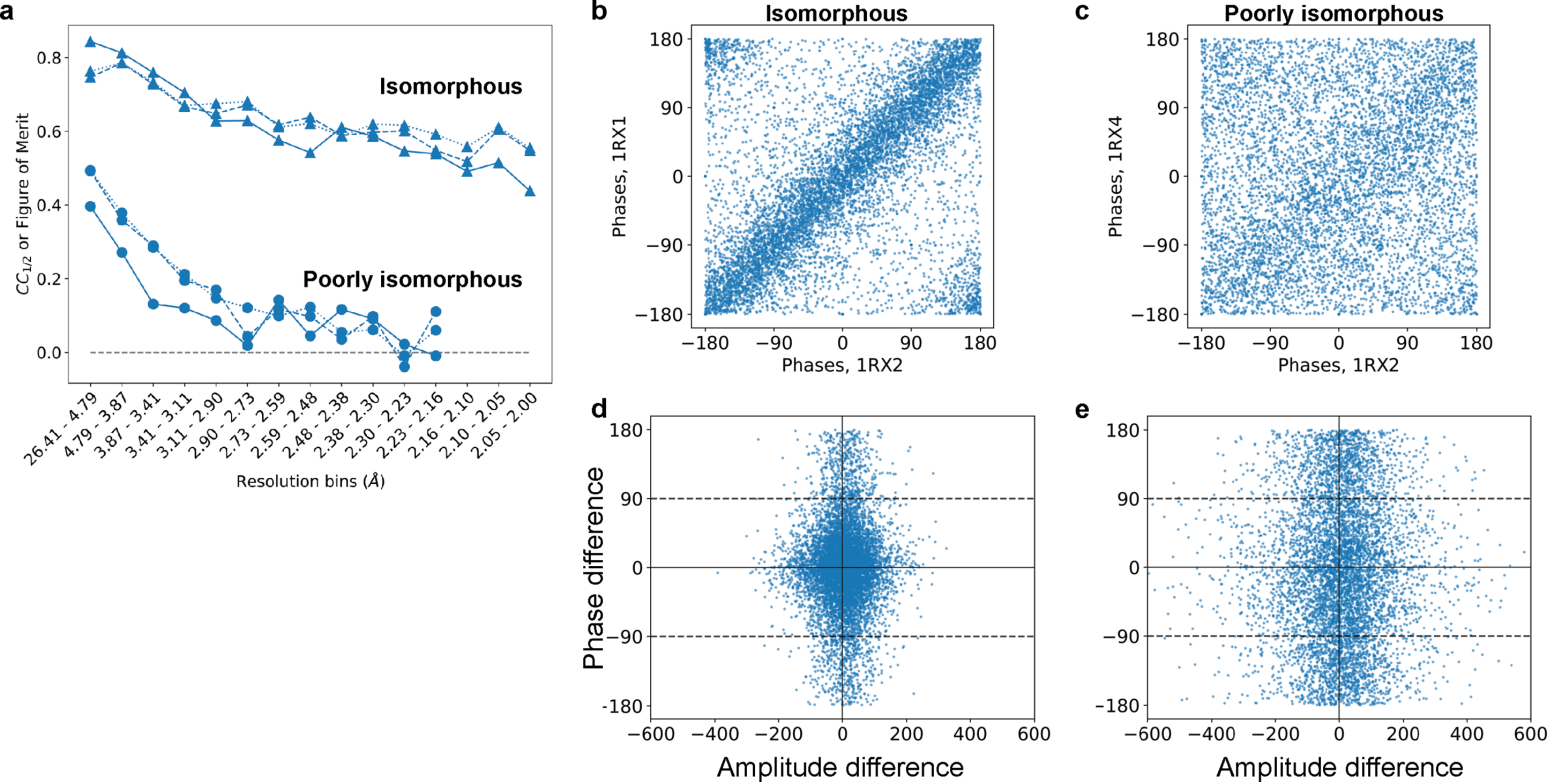
A demonstration of how structure factors depend sensitively on isomorphism. The *E. coli* DHFR data set 1rx2 is compared with a highly isomorphous structure (1rx1) and with a poorly isomorphous structure (1rx4, see also Fig. 2). (*a*) Correlation coefficients for the isomorphous pair (triangles) and poorly isomorphous pair (circles). Correlations were computed between structure factor amplitudes (solid lines) and cosines of structure factor phases (dashed lines), aggregated per resolution bin. A figure of merit (mean of cosine of differences) was computed between corresponding phases (dotted lines). While the isomorphous data correlate well even at high resolution, the poorly isomorphous data are uncorrelated even at moderate resolution. (*b*) and (*c*) Structure factor phases appear (*b*) highly correlated for the isomorphous structures but (*c*) mostly uncorrelated between poorly isomorphous structures. (*d*) and (*e*) For an isomorphous difference map to be meaningful, structure factor amplitudes should only differ when the phase difference is small, and structure factor phases should only differ when the amplitude difference is small (Rould & Carter, 2003 ▸). This requirement is met (*d*) in the isomorphous case but not (*e*) in the non-isomorphous case. In all panels, computed phases are obtained from the ‘PHIC’ column of the deposited MTZ files.

We begin by demonstrating the consequences of small deviations from perfect isomorphism. Our example makes use of three data sets (Sawaya & Kraut, 1997 ▸), all of *Escherichia coli* dihydrofolate reductase (DHFR) crystallized in space group *P*2_1_2_1_2_1_. These data sets vary by which ligands are bound to DHFR; we will discuss these ligands further below. Data sets 1rx2 and 1rx1 have unit-cell dimensions identical to within 0.4%, whereas data sets 1rx2 and 1rx4 differ by 2% along the *c* axis. Reflections in diffraction experiments report on different 3D frequency components of the electron density of molecules in the unit cell. As such, the shape of the molecular arrangement may look essentially the same (that is, isomorphous) at low spatial resolution yet entirely different at high resolution [recall that the contributions of different atoms *j* to structure factors add up by terms 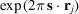 for scattering vector **s** and atomic position **r**_*j*_]. Measuring this quantitatively, we see much higher correlations between the structure factor amplitudes for our highly isomorphous pair of data sets (1rx2 and 1rx1) than for our ‘poorly isomorphous’ pair (1rx2 and 1rx4) [solid lines in Fig. 1 ▸(*a*)]. We find a similarly stark difference in correlations for the phases of refined models, whether measured by a figure of merit, 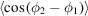 , or by a correlation coefficient (liable to small phase-wrapping artifacts). The loss in similarity of phases is visually striking [Figs. 1 ▸(*b*) and 1 ▸(*c*)].

We expect the consequences of such a loss of isomorphism to be severe: the computation of an isomorphous difference map requires that (i) amplitude differences are large only when phase differences are small, and conversely that (ii) phase differences are large only when amplitude differences are small. These requirements follow from equation (1) above and are depicted visually in the work of Rould & Carter (2003 ▸). The isomorphous data meet these requirements [Fig. 1 ▸(*d*)]. In contrast, the poorly isomorphous data sets display consistently large structure factor amplitude differences, regardless of the corresponding structure factor phase difference [Fig. 1 ▸(*e*)].

### 1.2. Rethinking isomorphous difference maps via the linearity of the Fourier transform

An isomorphous difference map is typically computed by first subtracting the structure factor amplitudes (*i.e.* sub­tracting in reciprocal space) and then applying the Fourier transform to convert the structure factor differences into a real-space difference map. However, because the Fourier transform and subtraction are both linear operations, their order can be switched without changing the result; one might just as well calculate two electron-density maps first and then subtract those maps voxel by voxel to yield an isomorphous difference map.

This reordering suggests how difference-map computation can be generalized beyond the isomorphous case. In particular, we see that the step in the algorithm most specific to the assumption of isomorphism is the construction of ‘hybrid’ structure factors, which combine the observed structure factor amplitudes for the ON data ( 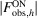 ) with the calculated structure factor phases for the OFF data ( 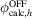 ). The resulting structure factors thus have the form 

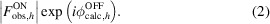

Critically, if the ON and OFF data differ in unit-cell volume and/or molecular orientation, these OFF phases may be incompatible with the ON amplitudes.

The method presented below improves these hybrid structure factors by computing phases that account for the (generally uninteresting) shifts in molecular position and orientation without removing any signal associated with ‘interesting’ changes.

## 2. The *MatchMaps* algorithm

The goal of *MatchMaps* is to achieve the best possible real-space difference density map without utilizing a prior model of any structural changes of interest. To compute a real-space difference density map, one first needs to approximate structure factor phases for each data set. As discussed above, the isomorphous difference map makes the simplifying assumption that the same set of structure factor phases can be used for both structures.

The key to *MatchMaps* is to improve phases for the ON data via rigid-body refinement of the OFF starting model against the ON structure factor amplitudes. This rigid-body refinement step improves phases by optimally placing the protein model in space. Critically, the restriction of this refinement to only whole-model rigid-body motion protects these new phases from bias towards modeled structural changes. The result is two sets of complex structure factors which make use of the information encoded in the structure factor amplitudes without relying on a second input model.

Next, each set of complex structure factors is Fourier-transformed into a real-space electron-density map. These two real-space maps will not necessarily overlay in space. However, the rotation and translation necessary to overlay the maps can be obtained from the results of the rigid-body refinement. Following real-space alignment, the maps can be subtracted voxel-wise to compute a difference map.

In the idealized case – similar structures, oriented identically in space, with identical unit cells – *MatchMaps* will perform essentially identically to an isomorphous difference map. However, as we show in the examples below, *MatchMaps* is more capable than a traditional isomorphous difference map of handling data sets that diverge from this ideal. Furthermore, even in seemingly simple cases where iso­morphous difference maps perform well, the real-space *MatchMaps* approach can show distinct improvements.

### 2.1. Details of algorithmic implementation

The full *MatchMaps* algorithm is as follows. As inputs, the algorithm requires two sets of structure factor amplitudes (referred to as ON and OFF data sets, for simplicity) and a single starting model (corresponding to the OFF data).

(i) If necessary, place both sets of structure factor amplitudes on a common scale using the *SCALEIT* (Henderson & Moffat, 1971 ▸) utility in *CCP4* (Agirre *et al.*, 2023 ▸).

(ii) Truncate both data sets to the same resolution range. This prevents the final difference map from preferentially displaying high-resolution features from the higher-resolution data set.

(iii) Generate phases for each data set via the *phenix.refine* program (Liebschner *et al.*, 2019 ▸). For each data set, the OFF starting model is used and only rigid-body refinement is permitted, to prevent the introduction of model bias. Bulk-solvent scaling may be either included (by default) or omitted from refinement. Including bulk-solvent scaling leads to better refinement statistics and higher map quality overall. However, bulk-solvent scaling may ‘flatten’ desired signal in the solvent region, *e.g.* for a large bound ligand. This trade-off is left to the user.

(iv) Create complex structure factors by combining observed structure factor amplitudes with computed structure factor phases obtained from refinement. Fourier transform each set of complex structure factors into a real-space electron-density map; this is performed using the Python packages *reciprocalspaceship* (Greisman *et al.*, 2021 ▸) and *gemmi* (Wojdyr, 2022 ▸).

(v) Compute the translation and rotation necessary to overlay the two rigid-body refined models. Apply this translation–rotation to the ON real-space map such that it overlays with the OFF map. These computations are carried out using *gemmi*. Note that the two rigid-body refined models are identical aside from translation and rotation, rendering trivial the atom selection for alignment.

(vi) Subtract real-space maps voxel-wise.

(vii) Apply a solvent mask to the final difference map.

We note that *MatchMaps* is structured such that Step (ii) can be generalized to not only rigid-body refinement but refinement of any ‘uninteresting features’ if the user provides a custom *PHENIX* (Liebschner *et al.*, 2019 ▸) parameter file as specified in the online documentation. For example, if the starting model contains multiple protein chains, each chain can be rigid-body-refined separately.

### 2.2. Installation

*MatchMaps* can be installed using the pip Python package manager (pip install matchmaps). The various pure-Python dependencies of *MatchMaps* are handled by pip. Additionally, *MatchMaps* requires installation of the popular *CCP4* and *PHENIX* software suites for crystallography. Once installed, the above protocol can be run in a single step from the command line.

In addition to the base *MatchMaps* command-line utility, the utilities matchmaps.ncs and matchmaps.mr provide additional functionalities explored in the examples below and the online documentation. *MatchMaps* is fully open source and readily extensible for novel use cases.

For more information, read the *MatchMaps* documentation at https://rs-station.github.io/matchmaps.

## 3. *MatchMaps* in the context of refinement and alternative approaches

*MatchMaps* is not a replacement for automatic and manual structural refinement of crystallographic data. Rather, we argue that *MatchMaps* provides a valuable supplement to structural refinement when the crystallographer seeks to characterize a structural change. *MatchMaps* can be implemented near the beginning of the analysis process to visualize the ON–OFF signal before an ON model has been refined. *MatchMaps* can also be used during or following refinement to validate or justify structural differences modeled during refinement of the ON and OFF models.

Below, we discuss two alternative methods which supplement structure refinement and which contrast interestingly with *MatchMaps*.

### 3.1. *F*_o_–*F*_c_ difference maps across data sets

A common element of structure refinement is the *F*_o_–*F*_c_ map (or, more precisely, *mF*_o_–*DF*_c_), which is used to describe how the modeled structure differs from the data. Details of the construction of such a map can be found elsewhere (Lamb *et al.*, 2015 ▸). In practice, *F*_o_–*F*_c_ maps are often the output of a procedure including refinement of atomic coordinates. In principle, however, an *F*_o_–*F*_c_ map can derive from a rigid-body-only refinement of a known structure to a new data set. In this latter scenario, the *F*_o_–*F*_c_ map is similar to a *MatchMaps* difference map (or, in an isomorphous case, to an isomorphous difference map).

**Figure 2:**
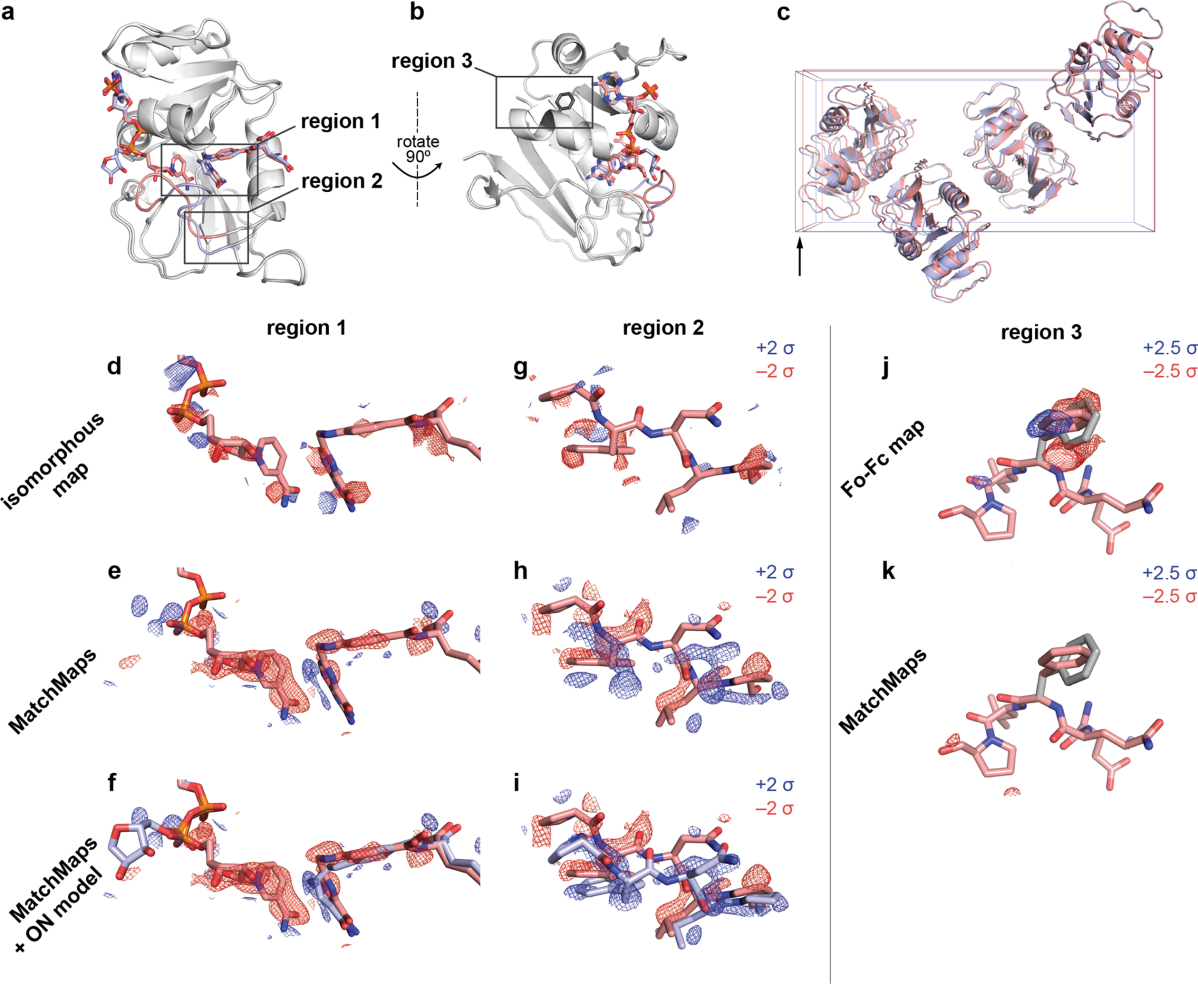
*MatchMaps* results for poorly isomorphous DHFR data sets. (*a*) The structures 1rx2 and 1rx4 are similar overall (gray cartoons). The structures differ mainly at the active-site loop (1rx2, red cartoon; 1rx4, blue cartoon) and in the positions of the active-site ligands (1rx2, red sticks; 1rx4, blue sticks). (*b*) The same as panel (*a*), but rotated 90° to the right about the vertical axis (indicated). The side chain for phenylalanine 103 is shown as dark-gray sticks. (*c*) The unit cells of 1rx2 and 1rx4 differ by 2% along the longest dimension (left to right in this figure) from 98.912 to 100.879 Å. (*d*)–(*f*) Visualizations of the change in ligand position between 1rx2 (red sticks) and 1rx4 [(*f*), blue sticks]. Positive difference density is shown as a blue mesh and negative difference density as a red mesh. Importantly, the 1rx4 structural coordinates were not used in the creation of the isomorphous or *MatchMaps* maps. The isomorphous difference map contains essentially no interpretable signal. In contrast, the *MatchMaps* map in panels (*e*) and (*f*) contains clear signal for disappearance of the cofactor and lateral sliding of the substrate. There is also faint positive signal associated with the ‘swung-out’ ribose ring, seen on the far left of the panel. Panel (*f*) is the same as panel (*e*), with the addition of the 1rx4 structural coordinates as blue sticks. (*g*)–(*i*) Visualizations of the change in loop conformation between 1rx2 and 1rx4. Only protein residues 21–25 are shown. Coloring is as in panels (*d*)–(*f*). (*g*) Again, the isomorphous difference map is not interpretable. (*h*) and (*i*) The *MatchMaps* positive difference density clearly corresponds to the 1rx4 structural model, which was not used in the creation of the map. Panel (*i*) is the same as panel (*h*), with the addition of the 1rx4 structural coordinates as blue sticks. (*j*) and (*k*) The impact of a spurious conformer on *F*_o_–*F*_c_ and *MatchMaps* maps, respectively. The 1rx2 model for residues 101–105 is shown as red sticks. The spurious conformer for Phe103 is shown as gray sticks. (*j*) The *F*_o_–*F*_c_ map shows clear positive (blue) and negative (red) density, recognizing the erroneous conformer as a conformational change. (*k*) *MatchMaps* does not show difference density for the spurious conformer.

**Figure 3:**
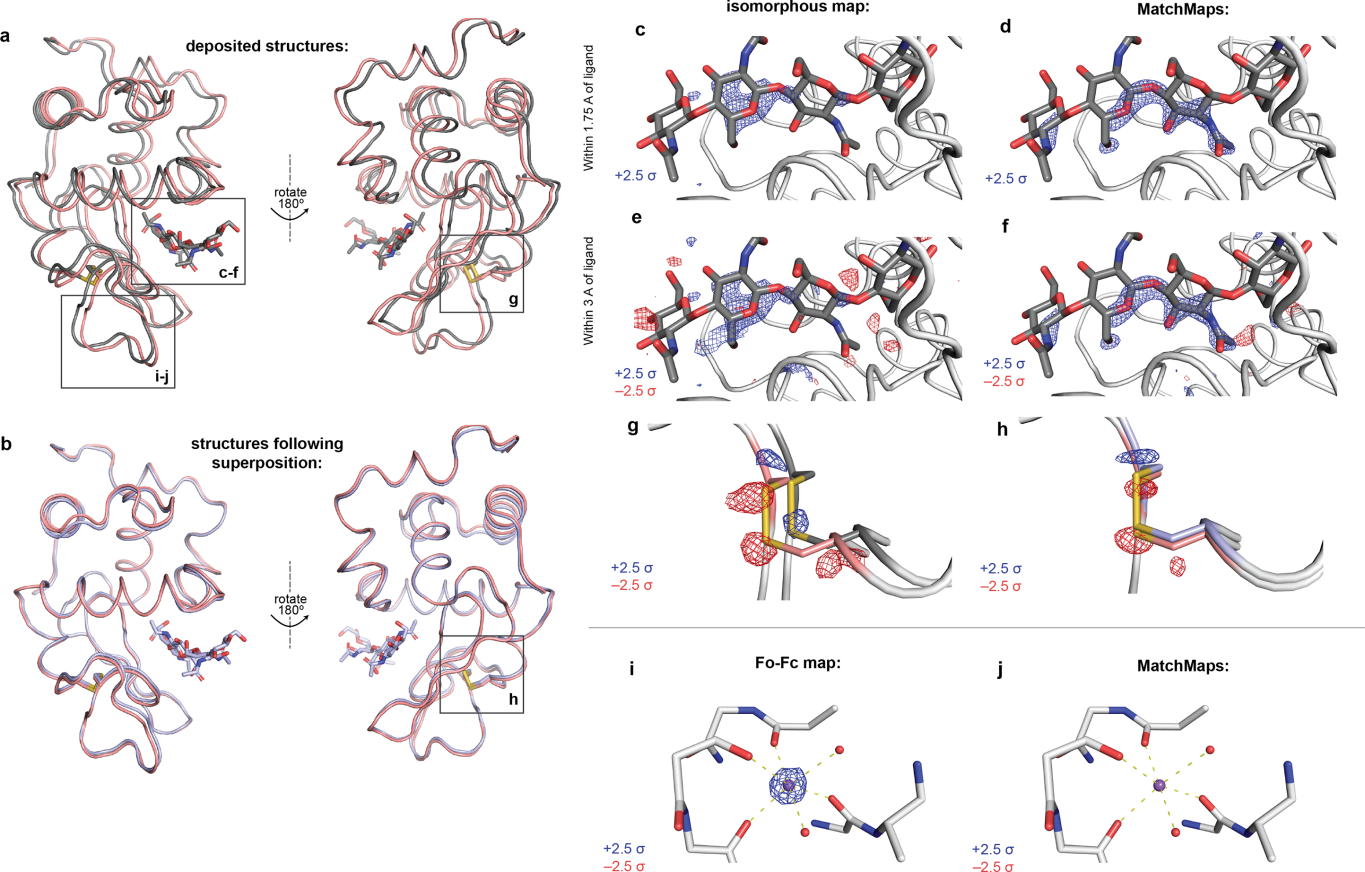
*MatchMaps* results for poorly isomorphous lysozyme data sets with a translation artifact. (*a*) Two views of the deposited coordinates for the ambient-pressure apo (4wld, red cartoon) and high-pressure GlcNAc-bound (4xen, gray cartoon, ligand as gray sticks) HEWL structures, which differ by a 1.48 Å translation (left to right from the view shown). (*b*) Two views following global alignment of the apo (red cartoon) and bound (blue cartoon, blue sticks) models, where the translation artifact disappears. Instead, the true structural change is revealed to be a slight constriction of the bound protein (up–down from the view shown). This constriction is 0.77 Å, as measured by the change in distance between Cα of residues 25 and 69. (*c*) An isomorphous difference map shows clear positive difference density (blue mesh, contoured at 2.5σ, carved at 1.75 Å from the ligand) around the ligand (gray sticks), but the density is weak and imprecise. (*d*) In contrast, *MatchMaps* at the same contour level (blue mesh, contoured at 2.5σ, carved at 1.75 Å from the ligand) shows high-resolution features of the ligand. (*e*) Carving the isomorphous map within 3 Å of the ligand reveals significant patches of both positive (blue) and negative (red) electron density with no clear structural correspondence. (*f*) The same visualization shows that *MatchMaps* is less noisy in this region. (*g*) Residues Cys64 and Cys80 are shown in red (apo, original coordinates) or dark gray (bound, original coordinates), with sulfur shown in yellow and other protein backbone in light gray. The positive (blue) and negative (red) isomorphous difference signals at 2.5σ correspond to the translation artifact between the unaligned coordinates. (*h*) Superposition of the bound model with the apo model (cysteines shown in blue). The *MatchMaps* density at 2.5σ is weaker and corresponds to the subtle vertical shift between the aligned structures. (*i*) and (*j*) Both structures contain a well-coordinated bound sodium ion. The sodium ion is shown as a purple sphere, the coordinating protein and water from the apo structure are shown as light-gray sticks, and hydrogen bonds are shown in yellow. (*i*) An *F*_o_–*F*_c_ difference map computed using the bound structure factor amplitudes and the apo model, but with the sodium ion omitted. This map erroneously suggests that the apo and bound data differ at the position of the sodium ion, when in fact the signal derives from modeling error. (*j*) *MatchMaps* shows no signal for this modeling error, as desired.

The difference between an *F*_o_–*F*_c_ map and a *MatchMaps* difference map is that, whereas *MatchMaps* only ever uses *observed* structure factor amplitudes, the *F*_o_–*F*_c_ map describes the OFF/reference data set using *calculated* structure factor amplitudes. In the limiting case where the OFF model describes the OFF data perfectly, the *F*_o_–*F*_c_ map should look like a *MatchMaps* difference map. In fact, an *F*_o_–*F*_c_ map may look better, because the map coefficients include only one set of measurement errors. Unfortunately, however, any modeling errors of the OFF/reference state will be included in the final *F*_o_–*F*_c_ map. Accordingly, in an *F*_o_–*F*_c_ map, it is impossible to distinguish ‘real signal’ (differences between the ON and OFF data) from modeling errors. We illustrate this undesired behavior below [Figs. 2(*j*)–2(*k*) and 3(*i*)–3(*j*)].

Note that the map coefficients for an *F*_o_–*F*_c_ map are created and saved by *MatchMaps* (if the --keep-temp-files flag is used), facilitating easy comparison between these two map types if desired.

### 3.2. PanDDA

A popular recent method for extracting subtle ligand-binding signal from crystallographic data is the pan-data-set density analysis (*PanDDA*) approach (Pearce *et al.*, 2017 ▸). A key practical difference between *PanDDA* and *MatchMaps* is that, while *PanDDA* expects several (typically of the order of dozens of) data sets, *MatchMaps* supports only two data sets at once. Additionally, whereas *MatchMaps* never changes the internal atomic coordinates of the input model, *PanDDA* aligns all input structures and maps via a local warping procedure. Thus, *PanDDA* reduces its ability to describe protein conformational changes in order to maximize its ability to detect weak ligand-binding events.

## 4. Examples

The following examples explore the benefits and functionalities offered by *MatchMaps*. All examples make use of published crystallographic data available from the Protein Data Bank (https://www.rcsb.org/). Scripts and data files for reproducing the figures can be found on Zenodo (Brookner & Hekstra, 2024 ▸).

### 4.1. *MatchMaps* for poorly isomorphous DHFR data sets

The enzyme dihydrofolate reductase is a central model system for understanding the role of conformational change in productive catalytic turnover (Sawaya & Kraut, 1997 ▸; Boehr *et al.*, 2006 ▸; Bhabha *et al.*, 2011 ▸). Specifically, the active-site Met20 loop of *E. coli* DHFR can adopt several different conformations, each stabilized by particular bound ligands and crystal contacts (Sawaya & Kraut, 1997 ▸). DHFR bound to NADP^+^ and substrate analog folate adopts a ‘closed’ Met20 loop (PDB ID 1rx2), whereas DHFR bound to NADP^+^ and product analog (dideaza­tetra­hydro­folate) adopts an ‘occluded’ Met20 loop (PDB ID 1rx4). These structures are highly similar, other than the relevant changes at the active site [Fig. 2 ▸(*a*), structural changes shown in boxes; r.m.s.d. 0.37 Å for protein Cα atoms excluding the Met20 loop].

Importantly, the presence of the occluded-loop conformation leads to altered crystal packing wherein the crystallographic *b* axis increases by 2%, from 98.91 to 100.88 Å [Fig. 2 ▸(*c*)]. Thus, 1rx2 and 1rx4 are ‘poorly isomorphous’, meaning that these structures, though extremely similar, cannot be effectively compared by an isomorphous difference map [Figs. 2 ▸(*d*) and 2 ▸(*g*)]. We illustrate the striking change in phase between these structures in Fig. 1 ▸. *MatchMaps* is able to account for this poor isomorphism and recover the expected difference signal.

First, we focus on ligand rearrangement in the active site. In the occluded-loop structure, the cofactor [Figs. 2 ▸(*d*)–2 ▸(*f*), left] leaves the active site while the substrate [Figs. 2 ▸(*d*)–2 ▸(*f*), right] slides laterally within the active site. *MatchMaps* shows this expected signal, with negative (red) difference density for the cofactor and paired positive (blue) and negative (red) difference density for the substrate [Figs. 2 ▸(*e*)–2 ▸(*f*)]. There is even faint positive signal for the ‘swung-out’ cofactor [Figs. 2 ▸(*e*)–2 ▸(*f*), far left]. By contrast, an isomorphous difference map [Fig. 2 ▸(*d*)] is unable to recover this signal. A model of the occluded-loop structure is shown for clarity in Fig. 2 ▸(*f*) as blue sticks and clearly matches the positive difference density. Importantly, this ON model is never used in the computation of the *MatchMaps* map.

We find a similar result around residues 21–25 of the Met20 loop [Figs. 2 ▸(*g*)–2 ▸(*i*)]. Again, *MatchMaps* shows readily interpretable difference signal for the change in loop conformation between the closed-loop (red) and occluded-loop (blue) structures [Figs. 2 ▸(*h*)–2 ▸(*i*)]. The isomorphous difference map, on the other hand, contains no interpretable signal in this region of strong structural change [Fig. 2 ▸(*g*)]. The occluded-loop model is shown for visual comparison in Fig. 2 ▸(*i*) but was not used for computation of the *MatchMaps* map.

#### 4.1.1. *MatchMaps* is not susceptible to modeling errors

As discussed above, *F*_o_–*F*_c_ maps can often display similar information to *MatchMaps* difference maps. However, *F*_o_–*F*_c_ maps will also contain signal that is not a difference between ON and OFF data sets, but rather results from modeling errors of the OFF model to the OFF data. We demonstrate this behavior by introducing a spurious conformer of phenylalanine 103 to the OFF starting model used above. Phe103 lies in a region distal to the ligands and active site [Fig. 2 ▸(*b*)]. An *F*_o_–*F*_c_ map, which inherently includes modeling errors, shows strong positive and negative difference density suggesting the correct Phe103 conformer [Fig. 2 ▸(*j*)]. From the *F*_o_–*F*_c_ map alone, it would be impossible to determine if this signal represented a difference between the ON and OFF data or a modeling error. In contrast, the *MatchMaps* difference map shows no difference density for this side chain [Fig. 2 ▸(*k*)]. This is the desired and expected result; neither data set’s *F*_o_ contains any information about this spurious conformer.

### 4.2. *MatchMaps* for poorly isomorphous HEWL data sets with a translation artifact

Hen egg-white lysozyme (HEWL) is among the best characterized model enzymes and has been the subject of many crystallographic analyses. One such analysis is high-pressure protein crystallography (HPPX), wherein crystal structures are collected at pressures ranging from ambient to hundreds of megapascals. Notably, HPPX is frequently associated with unit-cell changes. Here, we use *MatchMaps* to compare an ambient-pressure apo structure of HEWL (PDB ID 4wld) with a (GlcNAc)_4_-bound structure collected at 920 MPa (PDB ID 4xen) (Yamada *et al.*, 2015 ▸). The *a* and *b* axes of the unit cell shrink from 79.197 to 76.152 Å as a result of pressure, a change of nearly 4%.

First, we examine the positive difference density (blue mesh) for the bound (GlcNAc)_4_ (gray sticks) in both *MatchMaps* and an isomorphous difference map. While signal for the ligand is present in both maps, the density from *MatchMaps* is more clearly contoured to the high-resolution features of the ligand [Fig. 3 ▸(*d*)], whereas the isomorphous signal is weaker and less precisely located [Fig. 3 ▸(*c*)]. When viewing the density in the surrounding region at the same contour level (±2.5σ, positive as blue mesh, negative as red mesh), it is clear that the isomorphous map [Fig. 3 ▸(*e*)] is noisier than *MatchMaps* [Fig. 3 ▸(*f*)].

Additionally, these data illustrate how poor isomorphism can manifest as a strong translation artifact [Fig. 3 ▸(*a*)]. In this case, the main ‘interesting’ difference between the high- and low-pressure structures is a slight overall constriction of the protein. This change can be visualized by examining the structural models following alignment [Fig. 3 ▸(*b*)]. Relative to the low-pressure model (red cartoon), the high-pressure model (blue cartoon) moves downward in the upper half of the protein and upward in the lower half of the protein. This total constriction is 0.77 Å, measured as the change in distance between the Cα of residues 25 and 69. However, this subtle change is obscured when viewing the original unaligned coordinates from each structure [Fig. 3 ▸(*a*)]. The high-pressure model (gray cartoon) differs from the low-pressure model (red cartoon), not only by a slight constriction but also by a larger (1.48 Å) lateral translation.

By construction, isomorphous difference maps are susceptible to the translation artifact described here, whereas *MatchMaps* is not. This effect is visible throughout the isomorphous difference map, which is dominated by this artifact. As an example, we show the difference densities around the disulfide bond between Cys64 and Cys80. The positive (blue) and negative (red) signal in the isomorphous difference map [Fig. 3 ▸(*g*)] corresponds to the original unaligned coordinates from the low-pressure (red) and high-pressure (gray) models. In contrast, the positive and negative signal from *MatchMaps* [Fig. 3 ▸(*h*)] corresponds to the slight shift between the low-pressure model (red) and the high-pressure model (blue) following alignment to the low-pressure model.

#### 4.2.1. *MatchMaps* is not susceptible to modeling errors

The high-pressure data set again illustrates how modeling errors (differences between the OFF model and OFF data) will appear in an *F*_o_–*F*_c_ map derived from rigid-body refinement of the OFF model against the ON data. To illustrate this, we erroneously omitted a bound sodium ion. As expected, the *F*_o_–*F*_c_ map [Fig. 3 ▸(*i*)] shows strong positive (blue mesh) signal around the omitted sodium ion (purple sphere). Importantly, although this signal corresponds to a modeling error, it is indistinguishable from ‘real’ signal, *i.e.* a situation wherein the ion were present in the high-pressure structure but not the low-pressure structure. *MatchMaps* [Fig. 3 ▸(*j*)] does not display any signal for this ion, which is the desired behavior. Omitting the sodium ion from the OFF model has no significant effect on the *MatchMaps* signals described above.

### 4.3. *MatchMaps* for isomorphous PTP1B data with a rotation artifact

The enzyme protein tyrosine phosphatase 1B (PTP1B) plays a key role in insulin signaling (Elchebly *et al.*, 1999 ▸), making it a long-standing target for the treatment of diabetes using ortho- and allosteric drugs (Wiesmann *et al.*, 2004 ▸; Keedy *et al.*, 2018 ▸; Choy *et al.*, 2017 ▸). For illustration, we compare recent high-quality room-temperature structures of the apo protein (PDB ID 7rin) with the protein bound to the competitive inhibitor TCS401 (PDB ID 7mm1) (Greisman *et al.*, 2022 ▸). In addition to the presence/absence of signal for the ligand itself, the apo structure exhibits an equilibrium between ‘open’ and ‘closed’ active-site loops (Whittier *et al.*, 2013 ▸), whereas the bound structure shows only the closed loop.

**Figure 4:**
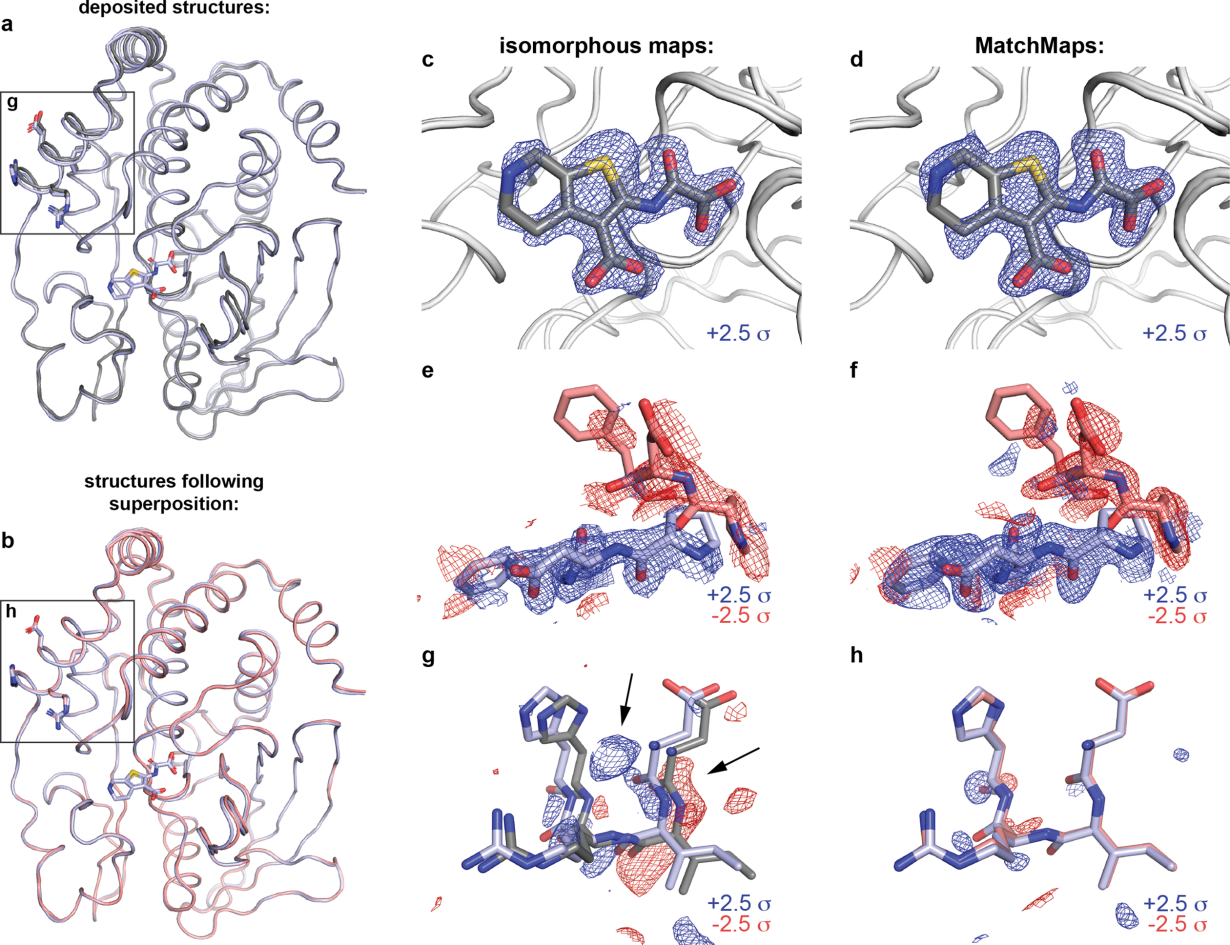
*MatchMaps* results for isomorphous PTP1B data with a rotation artifact. Comparison of the apo (7rin) and TCS401-bound (7mm1) structures of protein tyrosine phosphatase 1B. (*a*) The apo (gray) and bound (blue) structural models overlay well, but differ by a slight rotation. The difference in the models is especially apparent in the boxed region [see panel (*g*)]. (*b*) Aligning the apo model (red) to the bound model (blue) reveals that the structures overlay even better than the original coordinates suggest [see panel (*h*)]. (*c*) and (*d*) Both the isomorphous map (*c*) and the *MatchMaps* map (*d*) are clearly able to show the bound ligand. The TCS401 ligand (gray sticks) is shown for clarity but was not included in the computation of either map. Positive difference density is shown as blue mesh. (*e*) and (*f*) Close-ups of residues 180–182. Similarly to panels (*c*) and (*d*), the change in loop equilibrium between open (red mesh, red sticks) and closed (blue mesh, blue sticks) is apparent in both maps. (*g*) and (*h*) Residues 22–25 are shown. Though these data sets meet the requirements for isomorphism, the refined protein models still differ by a slight rotation. (*g*) The isomorphous difference map recognizes the artifactual difference between 7mm1 (blue) and 7rin (gray) model locations, which manifests as strong difference signal. This artifact is comparable in magnitude to the ‘true’ signal in panels (*c*) and (*e*). (*h*) *MatchMaps* internally aligns the data before subtraction and therefore avoids this artifact. The bound model after alignment to the apo model is shown in red. At ±2.5σ, there is no significant signal in the *MatchMaps* map for this region.

The data sets 7rin and 7mm1 are sufficiently isomorphous that an isomorphous difference map reveals the main structural changes. *MatchMaps* performs similarly. Strong positive difference density (blue mesh) is seen for the TCS401 ligand (gray sticks) in both the isomorphous difference map [Fig. 4 ▸(*c*)] and the *MatchMaps* difference map [Fig. 4 ▸(*d*)]. Around residues 180–182 of the active-site loop (known as the WPD loop), both the isomorphous difference map [Fig. 4 ▸(*e*)] and the *MatchMaps* difference map [Fig. 4 ▸(*f*)] show strong signal for a decrease in occupancy (red mesh) of the open-loop conformation (red sticks) and an increase in occupancy (blue mesh) of the closed-loop conformation (blue sticks).

However, even in this seemingly straightforward case, we find that the isomorphous difference map is susceptible to an artifact resulting from a slight (1.37°) rotation of the protein. The displacement between the original refined structural coordinates of each structure is especially strong around residues 22–25 [Fig. 4 ▸(*a*), boxed region; Fig. 4 ▸(*g*), apo model in gray, bound model in blue]. In this region, an isomorphous difference map picks up on this artifactual difference between the data sets and displays strong difference signal (blue and red mesh). Remarkably, this signal is similar in magnitude to the ‘true’ signal seen in Figs. 3 ▸(*c*) and 3 ▸(*e*). In contrast, *MatchMaps* internally aligns the data before subtraction of electron density. Fig. 4 ▸(*b*) (boxed region) and Fig. 4 ▸(*h*) (apo model in red, bound model in blue) show residues 22–25 following whole-molecule alignment of the protein models. Following global alignment of the refined models, it is clear that this region does not contain significant ‘interesting’ signal. Sure enough, the *MatchMaps* difference map contains no strong signal in this region. In fact, the faint signal that persists in the *MatchMaps* map for this region seems to suggest a slight remaining coordinate displacement in this region following whole-molecule alignment.

### 4.4. matchmaps.mr for DHFR data from different space groups

For many protein systems, careful analysis of electron-density change is stymied for pairs of similar structures which crystallize in different crystal forms. The *MatchMaps* algorithm can be further generalized to allow comparison of data sets in entirely different crystal packings or space groups. Specifically, the OFF model can serve as a search model for molecular replacement for the ON data. Following this extra step, the algorithm proceeds identically. We implement this modified algorithm in the command-line utility matchmaps.mr.

**Figure 5:**
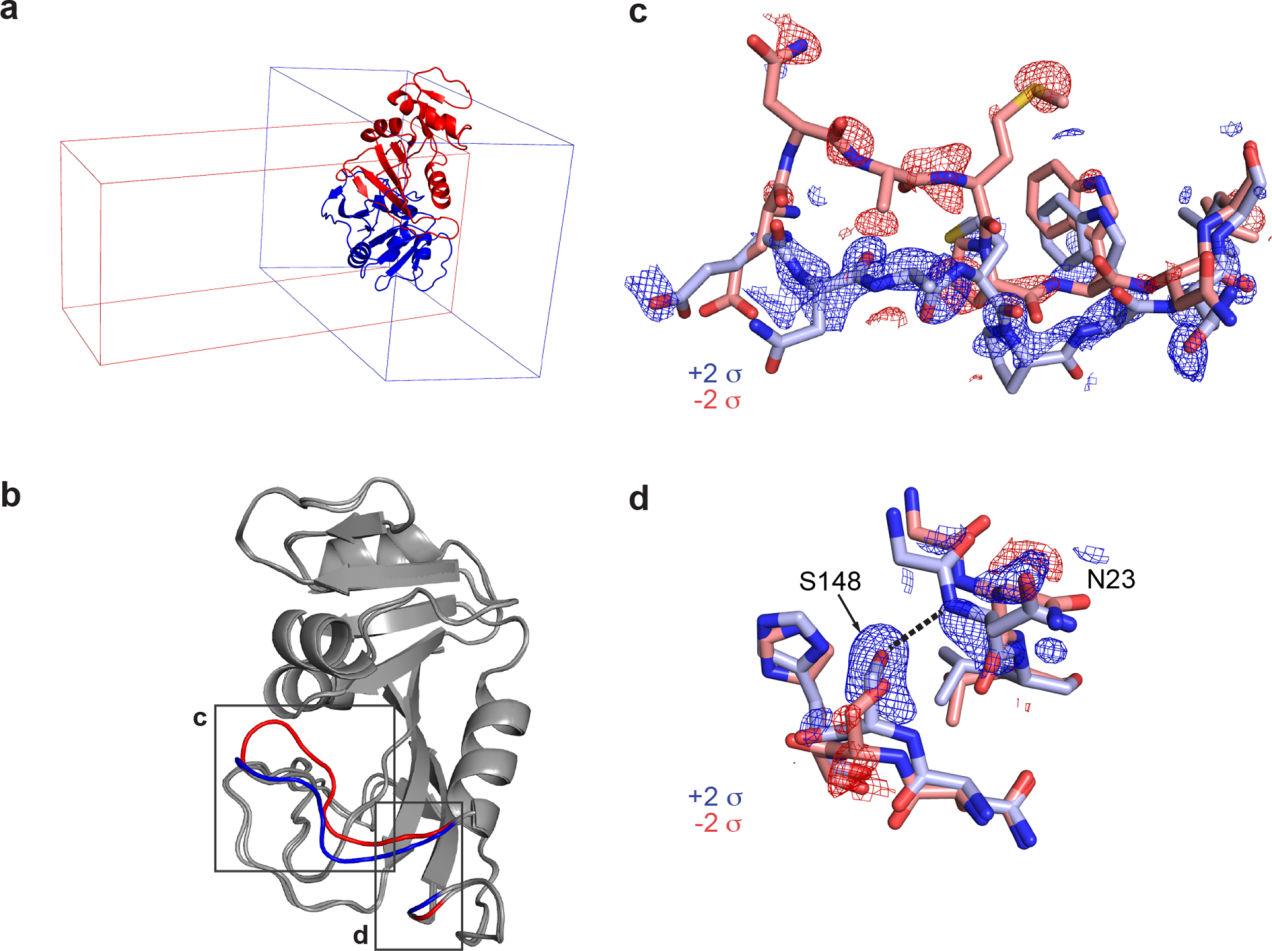
Results of matchmaps.mr for DHFR data from different space groups. A variant of *MatchMaps* (implemented in the command line as matchmaps.mr) can be used to compute difference maps between two crystallographic data sets in entirely different space groups. (*a*) An overlay of structural models of DHFR in space group *P*2_1_2_1_2_1_ (PDB ID 1rx1, blue cartoon) and space group *C*2 (PDB ID 1ra1, red cartoon), along with the respective unit cells for each. (*b*) Alignment of the structures in *P*2_1_2_1_2_1_ and *C*2 shows the global agreement of the structures. Structural differences are localized to the active site (boxed regions, *P*2_1_2_1_2_1_ structure in red, *C*2 structure in blue) and are known to result from differences in crystal packing. (*c*) A close-up on residues 17–24. The *MatchMaps* positive (blue) and negative (red) difference densities clearly correspond to the refined structural coordinates for the *P*2_1_2_1_2_1_ (red) and *C*2 (blue) models. Remarkably, the positive difference density is strong and clearly corresponds to the *C*2 structure, despite the *C*2 structure never being used in the creation of the map. (*d*) A close-up on the hydrogen bond between residues Ser148 and Asn23, which is only present in the *C*2 crystal form (blue sticks). The *MatchMaps* (positive) difference density clearly indicates the hydrogen-bond-capable conformation.

One such example is the enzyme DHFR, which has been crystallized in many space groups (Sawaya & Kraut, 1997 ▸). Here, we examine two structures of the enzyme bound to NADP^+^, in space groups *P*2_1_2_1_2_1_ (PDB ID 1rx1) and *C*2 (PDB ID 1ra1), visualized in Fig. 5 ▸(*a*). These structures are similar overall but differ in the active site [Figs. 5 ▸(*b*)–5 ▸(*d*)]. Here, we visualize these structural changes directly in electron density without introducing model bias.

Specifically, in the *P*2_1_2_1_2_1_ structure, the active-site Met20 loop adopts a closed conformation. In the *C*2 structure, the Met20 loop adopts an ‘open’ conformation, which is stabilized by a crystal contact in this crystal form (Sawaya & Kraut, 1997 ▸). The difference between the open and closed loops is exemplified by residues 17–24 [Fig. 5 ▸(*c*)]. The open loop is stabilized by the formation of a key hydrogen bond between the Asn23 backbone and the Ser148 side chain. In the closed conformation, Asn23 is too far from Ser148 to form a hydrogen bond [Fig. 5 ▸(*d*)].

Remarkably, the positive difference density (blue) for the open loop is strong and readily interpretable in Figs. 5 ▸(*c*)–5 ▸(*d*). The *MatchMaps* map was computed using only the *P*2_1_2_1_2_1_ (red) closed-loop model. This means that the signal for the open-loop conformation is derived only from the observed structure factor amplitudes for the open-loop state in an unrelated crystal form.

### 4.5. matchmaps.ncs for NCS-related molecules of PDZ

**Figure 6:**
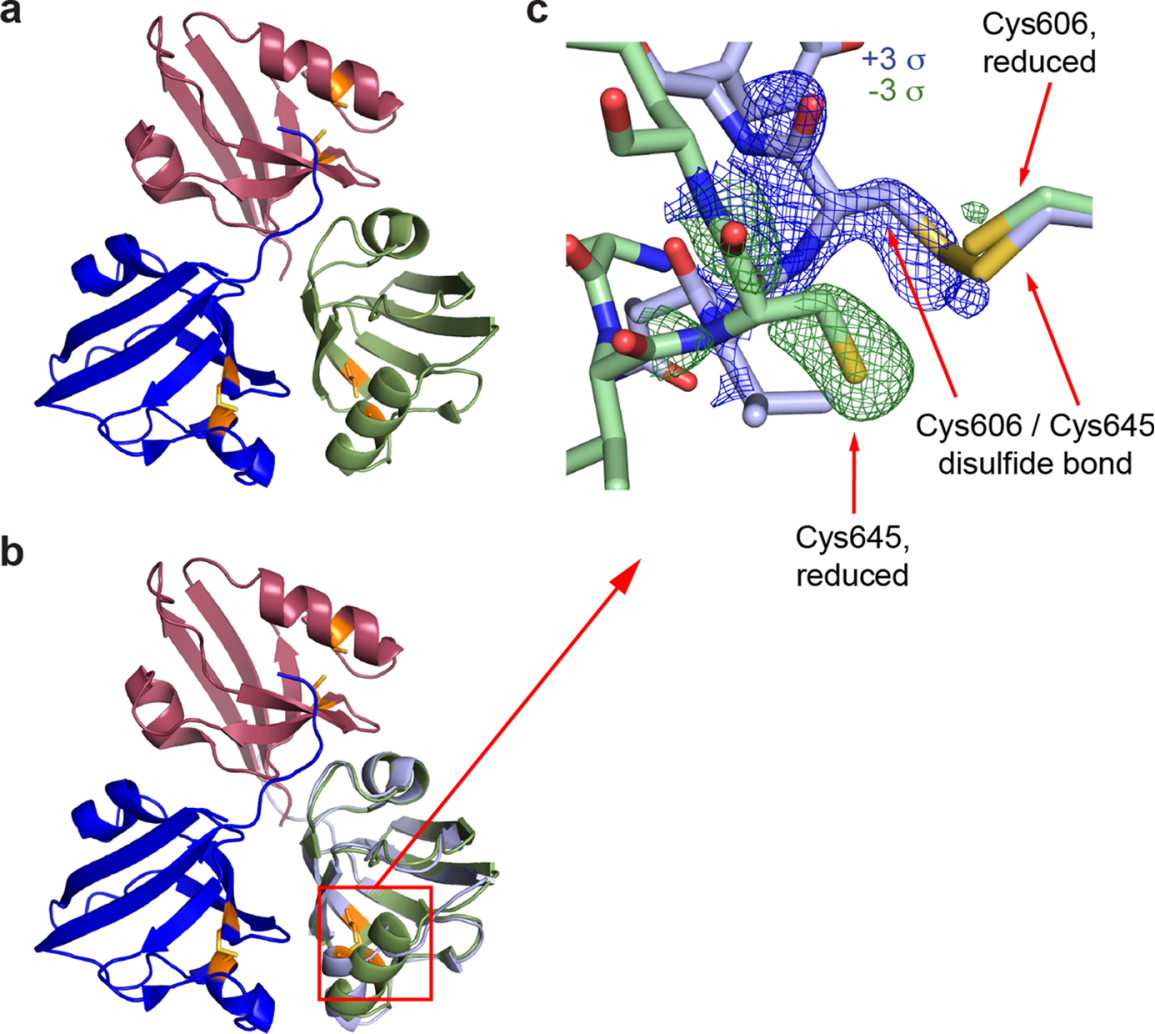
Results of matchmaps.ncs for NCS-related molecules of PDZ. A variant of *MatchMaps* (implemented in the command line as matchmaps.ncs) can be used to compute internal difference maps across an NCS operation. (*a*) An overview of the three PDZ domains related by non-crystallographic symmetry. Chain *A* is shown in red, chain *B* in green and chain *C* in blue. Residues Cys606 and Cys645, which can form a disulfide bond, are shown in orange. The coloring matches Fig. 2(*c*) from the report by Mishra *et al.* (2007 ▸). (*b*) The same as panel (*a*), plus a copy of chain *C*, shown in light blue, aligned and superimposed onto chain *B*. (*c*) A close-up on the disulfide bond formation. Chain *C* (light-blue sticks) contains a disulfide bond between Cys606 and Cys645, whereas chain *B* (green sticks) does not. The positive (blue) and negative (green) difference density corresponding to each chain is clearly visualized by *MatchMaps*.

The real-space portion of the *MatchMaps* algorithm can be repurposed to create ‘internal’ difference maps across non-crystallographic symmetry (NCS) operations. We implement this modified algorithm in the command-line utility matchmaps.ncs. As an example, we examined the crystal structure of the fifth PDZ domain (PDZ5) from the *Drosophila* protein Inactivation, no after-potential D (INAD). This domain plays an essential role in terminating the response of photoreceptors to absorbed photons by modulation of its ability to bind ligands (Mishra *et al.*, 2007 ▸). In particular, the binding cleft of PDZ5 can be locked by formation of a disulfide bond between residues Cys606 and Cys645. PDZ5 was found to crystallize in a form with three molecules in the asymmetric unit [Fig. 6 ▸(*a*)] where each molecule adopts a different state. Specifically, chain *C* contains a disulfide bond between residues Cys606 and Cys645, whereas chain *B* does not. Chains *B* and *C* overlay well other than the disulfide bond region [Fig. 6 ▸(*b*)]. Chain *A* adopts a bound state by binding the C terminus of chain *C* (not shown). *MatchMaps* enables calculation of an internal difference map, yielding a clearly interpretable difference map for the formation of the disulfide bond [Fig. 6 ▸(*c*)].

## 5. Discussion

The isomorphous difference map has been a popular method for detecting conformational change for many years (Henderson & Moffat, 1971 ▸; Rould & Carter, 2003 ▸). However, we have shown above that the same inputs – one structural model and two sets of structure factor amplitudes – can be combined to compute a difference map that shares the strengths of an isomorphous difference map while ameliorating a key weakness. Specifically, structure factor phases are highly sensitive not only to structural changes (‘interesting’ signal) but also to changes in unit-cell dimensions and model pose (‘un­interesting’ signal). The introduction of rigid-body refinement minimizes the contribution of this uninteresting signal to the final difference map. In Fig. 2 ▸, we illustrate a case where a loss of isomorphism significantly degrades the signal of an isomorphous difference map. In this case, *MatchMaps* is still able to recover the expected difference signal.

Changes in unit-cell volume frequently involve a dis­proportionate contribution from changes in solvent volume (Atakisi *et al.*, 2018 ▸; Yamada *et al.*, 2015 ▸), whereas the protein volume changes less. In such a situation, the protein location relative to the unit cell must change in some systematic way. This systematic change is an inherent part of the signal detected by an isomorphous difference map. We demonstrate in Figs. 3 ▸ and 4 ▸ that isomorphous difference maps are highly susceptible to translation and rotation artifacts, whereas *MatchMaps*, by virtue of construction, does not contain these artifacts. We emphasize that this problem with isomorphous difference maps is inherent and thus likely to be widespread.

In our experience, crystallographic perturbation experiments are often shelved due to changes in unit-cell constants. *MatchMaps* removes, in principle, the requirement for isomorphism and allows for the analysis of more crystallographic differences.

The computation of an isomorphous difference map is entirely incompatible with data from different crystal forms. The matchmaps.mr extension of *MatchMaps* allows for model-bias-free comparisons of electron densities regardless of crystal form, opening up a new world of structural comparisons. For instance, an isomorphous difference map cannot characterize the impacts of crystal packing. As shown above, *MatchMaps* can create such a map and thus allows enhanced understanding of the often subtle role of crystal packing on protein structure.

*MatchMaps* depends only on the common *CCP4* and *PHENIX* crystallographic suites, along with various automatically installed pure-Python dependencies. *MatchMaps* runs in minutes on a modern laptop computer. The only required input files are a PDB or mmCIF file containing the protein model, two MTZ files containing structure factor amplitudes and uncertainties, and any CIF ligand restraint files necessary for refinement. These are the same inputs as required for many common purposes (such as running *phenix.refine*) and would probably already be on hand. As outputs, *MatchMaps* produces real-space maps in the common MAP/CCP4/MRC format which can be readily opened in molecular visualization software such as *PyMOL* (https://pymol.org/) or *Coot* (Emsley *et al.*, 2010 ▸). For these reasons, *MatchMaps* should slot naturally into the crystallographer’s workflow for analysis of related data sets. Additionally, *MatchMaps* is open source and can be easily modified for a new use case by an interested developer. The authors welcome issues and pull requests on GitHub for the continued improvement of the software.

## Supplementary Material

data used; scripts for analysis and creating figures: https://doi.org/10.5281/zenodo.10452581
